# Fairness of Machine Learning Algorithms for Predicting Foregone Preventive Dental Care for Adults

**DOI:** 10.1001/jamanetworkopen.2023.41625

**Published:** 2023-11-03

**Authors:** Helena Silveira Schuch, Mariane Furtado, Gabriel Ferreira dos Santos Silva, Ichiro Kawachi, Alexandre D. P. Chiavegatto Filho, Hawazin W. Elani

**Affiliations:** 1 Harvard School of Dental Medicine, Harvard University, Boston, Massachusetts; 2 School of Public Health, University of Sao Paulo, Sao Paulo, Brazil; 3 Harvard T. H. Chan School of Public Health, Harvard University, Boston, Massachusetts

## Abstract

**Importance:**

Access to routine dental care prevents advanced dental disease and improves oral and overall health. Identifying individuals at risk of foregoing preventive dental care can direct prevention efforts toward high-risk populations.

**Objective:**

To predict foregone preventive dental care among adults overall and in sociodemographic subgroups and to assess the algorithmic fairness.

**Design, Setting, and Participants:**

This prognostic study was a secondary analyses of longitudinal data from the US Medical Expenditure Panel Survey (MEPS) from 2016 to 2019, each with 2 years of follow-up. Participants included adults aged 18 years and older. Data analysis was performed from December 2022 to June 2023.

**Exposure:**

A total of 50 predictors, including demographic and socioeconomic characteristics, health conditions, behaviors, and health services use, were assessed.

**Main Outcomes and Measures:**

The outcome of interest was foregoing preventive dental care, defined as either cleaning, general examination, or an appointment with the dental hygienist, in the past year.

**Results:**

Among 32 234 participants, the mean (SD) age was 48.5 (18.2) years and 17 386 participants (53.9%) were female; 1935 participants (6.0%) were Asian, 5138 participants (15.9%) were Black, 7681 participants (23.8%) were Hispanic, 16 503 participants (51.2%) were White, and 977 participants (3.0%) identified as other (eg, American Indian and Alaska Native) or multiple racial or ethnic groups. There were 21 083 (65.4%) individuals who missed preventive dental care in the past year. The algorithms demonstrated high performance, achieving an area under the receiver operating characteristic curve (AUC) of 0.84 (95% CI, 0.84-0.85) in the overall population. While the full sample model performed similarly when applied to White individuals and older adults (AUC, 0.88; 95% CI, 0.87-0.90), there was a loss of performance for other subgroups. Removing the subgroup-sensitive predictors (ie, race and ethnicity, age, and income) did not impact model performance. Models stratified by race and ethnicity performed similarly or worse than the full model for all groups, with the lowest performance for individuals who identified as other or multiple racial groups (AUC, 0.76; 95% CI, 0.70-0.81). Previous pattern of dental visits, health care utilization, dental benefits, and sociodemographic characteristics were the highest contributing predictors to the models’ performance.

**Conclusions and Relevance:**

Findings of this prognostic study using cohort data suggest that tree-based ensemble machine learning models could accurately predict adults at risk of foregoing preventive dental care and demonstrated bias against underrepresented sociodemographic groups. These results highlight the importance of evaluating model fairness during development and testing to avoid exacerbating existing biases.

## Key Points

**Question:**

Can machine learning (ML) predict foregone preventive dental care among adults, and is the ML model performance fair across sociodemographic subgroups?

**Findings:**

In this prognostic study including 32 234 adults, tree-based ensemble prediction models demonstrated high performance; however, the models performed worse for individuals from racial or ethnic minority groups, low-income, and younger adults.

**Meaning:**

In this prognostic study, the ML models accurately predicted foregone preventive dental care; the models had low precision in discriminating the outcome for underrepresented minority groups, highlighting the importance of evaluating algorithms’ fairness to avoid exacerbating existing biases.

## Teaser

This prognostic study uses machine learning to predict foregone preventive dental care among adults overall and in sociodemographic subgroups and assesses the algorithmic fairness.

## Introduction

Dental disease affects nearly 3.5 billion people worldwide.^1^ In the US, more than 90% of adults have dental caries, and nearly 50% of adults have gum disease.^2^ However, most oral health conditions are preventable if identified and treated early. Routine access to preventive dental services can prevent acute dental problems, such as pain, emergency department use, and advanced dental disease. Nevertheless, in 2022, more than 90 million adults (approximately 64.1% of the US adult population) did not have a dental visit in the past year.^3^

Disadvantaged socioeconomic groups and racial and ethnic minority groups, such as Black and Hispanic individuals, who face additional barriers to accessing dental care,^4^ such as limited transportation, lack of health insurance, and precarious employment,^5^ experience a disproportional share of dental diseases. Cost is the main barrier to accessing dental care,^6,7^ with Black and Hispanic adults more likely to face those barriers compared with Asian and White adults.^8^ Moreover, the risk of Black and low-income adults visiting an emergency department for dental conditions is significantly higher than for other sociodemographic groups.^7,9^ Identifying patients at risk of not accessing preventive dental care can direct resources and prevention efforts toward improving access and reducing inequities in dental care.

Machine learning (ML) approaches have the potential to improve health care delivery for disadvantaged populations.^10^ ML algorithms identify patterns from existing data through statistical modeling to predict future outcomes.^11,12^ However, if the algorithms are trained with biased data, they may end up reproducing disparities in access to care. Therefore, current best practices call for fairness-aware ML, focusing on designing fair algorithms and detecting and eliminating biases.^13^ In this study, we aim to predict foregone preventive dental care among adults and to examine the fairness of the algorithms for underrepresented sociodemographic groups.

## Methods

This prognostic study was determined to be not human research by the institutional board review of Harvard University and exempt from informed consent because we used publicly available, deidentified data. We followed the Transparent Reporting of a Multivariable Prediction Model for Individual Prognosis or Diagnosis (TRIPOD) reporting guideline.

### Data

We used data from the Medical Expenditures Panel Survey (MEPS). The MEPS is a set of panel surveys conducted by the US Agency for Healthcare Research and Quality (AHRQ) that includes information on the use of health care, health expenditures, and health insurance coverage. For this study, we used data from the longitudinal MEPS Household Component (MEPS-HC). The survey is designed to be representative of the US civilian noninstitutionalized population. The households included in MEPS-HC are a subsample of respondents of the National Health Interview Survey in the previous year. Hispanic individuals and non-Hispanic Black individuals are oversampled in National Health Interview Survey’s sample design. More information on the MEPS methodology is publicly available elsewhere.^14^

Each year, a new MEPS panel survey, with a new sample of households, is collected. Each household is evaluated 5 times (rounds) over 2 years. For this study, we used longitudinal data from MEPS panels 21 (2016-2017), 22 (2017-2018), and 23 (2018-2019). Participants were exclusive to each panel and, for this study, we combined all 3 cohorts. Therefore, our analytical data set included all adult participants in the MEPS from 2016 to 2019. Data analysis was performed from December 2022 to June 2023.

Our sample included adults ages 18 years and older. We examined the full sample and sociodemographic subgroups by age, income, and race and ethnicity. Age subgroups included working-age (18-64 years) and older (≥65 years) adults. We defined income subgroups based on the family income as a percentage of the federal poverty line, with low-income defined as less than 200% and high-income, 200% or more. We categorized race and ethnicity subgroups, based on self-reported data, as Hispanic, non-Hispanic Asian, non-Hispanic Black, non-Hispanic White, and non-Hispanic other race or multiple racial groups. The other category included groups that were too small to be evaluated individually, such as American Indian and Alaska Native.

### Predictors

We used 50 variables from the first year of each panel based on prior literature and subject-matter expertise. These variables included demographic and socioeconomic characteristics, health conditions, self-perceived health, behaviors, and health services use. Examples of demographic variables are age, sex, and marital status; and socioeconomic status characteristics included family income, education, and employment status. We included health conditions identified by the AHRQ as priority conditions: hypertension, heart disease, high cholesterol, emphysema, chronic bronchitis, diabetes, cancer, arthritis, asthma, and stroke.^15^ We also included disability and limitations variables and self-perceived health and behaviors, such as self-perceived general and mental health, smoking, and physical activity. Health services use included information on the number and type of visits and health care expenditures. Full variable descriptions are presented in eTable 1 in Supplement 1.

### Output

Our outcome of interest was foregoing preventive dental care, defined as not having a preventive procedure in the second year of the panel. Preventive procedures include cleaning, general examination, or an appointment with the dental hygienist. Details on output definition are presented in eTable 1 in Supplement 1.

### Preprocessing Techniques

We applied 1-hot encoding to preprocess categorical variables with more than 2 categories. We standardized continuous variables with *z*-scores and imputed missing values at the predictor’s median in the training set. To tune the hyperparameters, we used randomized search with 10-fold cross-validation and 30 iterations.^16^ Estimates are from algorithms without any balancing technique. However, due to the unbalanced nature of the data, in sensitivity analyses, we also implemented the synthetic minority over-sampling technique (SMOTE) in the training data set.^17^

We developed models using 4 commonly used ML algorithms for structured health data: extreme gradient boosting (XGB) (xgboost version 1.7.6),^18^ light gradient boosting (lightgbm version 2.3.1),^19^ catboost (catboost version 1.2.1),^20^ and random forest (RandomForestClassifier within scikit-learn library version 1.3.0).^21,22^ Models were trained with data from MEPS panels 2016 to 2018 and tested in panels 2018 to 2019.

### Statistical Analysis

We assessed the algorithms’ performances by examining the area under the receiver operating characteristic curve (AUC) and its 95% CI, sensitivity (recall), specificity, precision (positive predictive value), and F1 score (harmonic mean between sensitivity and precision).

To examine the algorithms’ fairness, we conducted analyses based on race and ethnicity, age, and family income subgroups. We tested 3 approaches.^23^ First, we examined the performance of the full sample model when applied to each sociodemographic subgroup. Second, we generated neutral models, by removing the subgroup-sensitive predictor.^24^ Third, we generated stratified models that were specific to each subgroup. CIs for applying the full sample model to sociodemographic subgroups were generated with 1000 bootstraps.

To examine the model’s ability in discriminating between classes of the output, we plotted the prediction density of each ML model. Finally, we performed feature importance analyses by calculating Shapley Additive Explanation (SHAP) values for the best performing algorithm. SHAP measures the importance of each input to the full sample model prediction performance.^25^ Analyses were conduced using Stata SE version 15.0 and Python version 3.8.12 programming language.

## Results

Our study included 32 234 adults, of whom 25 052 were aged 18 to 64 years and 7182 were 65 years and older (mean [SD] age, 48.5 [18.2] years) (Table 1). Women represented 53.9% of the sample (17 386 individuals). The study population included 1935 Asian participants (6.0%), 5138 Black participants (15.9%), 7681 Hispanic participants (23.8%), 16 503 White participants (51.2%), and 977 participants (3.0%) self-identified as other or multiple racial groups. A total of 21 083 individuals (65.4%) missed preventive dental care in the past year. Sample characteristics were similar in the training and testing data sets (eTable 2 in Supplement 1). Survey-weighted and unweighted samples were similar (eFigure 1 and eFigure 2 in Supplement 1).

**Table 1.** Description of the Study Sample

Characteristic	Adults, No. (%)^a^	
	Full sample (N = 32 234)	Missed preventive dental care (n = 21 083)
Panel		
2016-2017 (Training set)	11 174 (34.7)	7690 (36.5)
2017-2018 (Training set)	10 613 (32.9)	6778 (32.1)
2018-2019 (Test set)	10 447 (32.4)	6615 (31.4)
Sex		
Male	14 848 (46.1)	10 239 (48.6)
Female	17 386 (53.9)	10 844 (51.4)
Age, y		
Mean (SD)	48.5 (18.2)	47.0 (18.2)
18-64	25 052 (77.7)	16 950 (80.4)
≥65	7182 (22.3)	4133 (19.6)
Education		
<High school	6533 (20.4)	5408 (26.0)
High school diploma or GED	13 700 (42.9)	9716 (46.6)
Some college or college graduate	11 715 (36.7)	5711 (27.4)
Race and ethnicity		
Asian	1935 (6.0)	1228 (5.8)
Black	5138 (15.9)	4004 (19.0)
Hispanic	7681 (23.8)	6036 (28.6)
White	16 503 (51.2)	9113 (43.2)
Other^b^	977 (3.0)	702 (3.3)
Family income, % of federal poverty line		
<200	11 483 (35.6)	9185 (43.6)
200-399	9352 (29.0)	6388 (30.3)
≥400	11 399 (35.4)	5510 (26.1)
Heath insurance		
Uninsured	3299 (10.2)	2932 (13.9)
Public only	9090 (28.2)	6827 (32.4)
Any private	19 845 (61.6)	11 324 (53.7)
Dental insurance		
No	20 008 (62.2)	14 476 (68.8)
Yes	12 176 (37.8)	6566 (31.2)
Smoking	6274 (19.5)	4962 (23.6)
Medical condition		
Asthma	3966 (12.3)	2549 (12.1)
Arthritis	8493 (26.4)	5150 (24.4)
Bronchitis	737 (2.3)	551 (2.7)
Cancer	3363 (10.4)	1766 (8.4)
Diabetes	4016 (12.5)	2817 (13.4)
Emphysema	685 (2.1)	528 (2.5)
Heart diseases	4688 (14.6)	2925 (13.9)
Hypertension	11 215 (34.8)	7339 (34.8)
High cholesterol levels	9994 (31.0)	6046 (28.7)
Stroke	1412 (4.4)	1008 (4.8)

Abbreviation: GED, general education development.

^a^ Unweighted estimates from the Medical Expenditure Panel Survey, 2016 to 2019. All predictors collected in year 1.

^b^ Includes groups that were too small to be evaluated individually, such as American Indian and Alaska Native individuals, or those reporting multiple racial groups.

Table 2 presents the performance of the XGB models in predicting foregoing preventive dental care in the test data set. It reports on 3 approaches: the full sample model, generated with the full population and applied to each subgroup; the full sample model, generated without each of the sensitive attributes (ie, excluding race and ethnicity, age, and income); and models developed for each subgroup. The full sample model demonstrated high performance, with an AUC of 0.84 (95% CI, 0.84-0.85). The full sample model performed similarly when applied to White participants only and demonstrated the highest performance for older adults (AUC, 0.88; 95% CI, 0.87-0.90). However, we detected a loss of performance among all other subgroups. The worst performance was observed when applying the full sample model to Black adults (AUC, 0.78; 95% CI, 0.75-0.81). When using the standard threshold of 0.5, the models showed high sensitivity (range, 0.79-0.94) but were less specific (0.40-0.79) (Table 2). When defining the threshold according to the proportion of the outcome in each sample, the models were more specific but less sensitive (Table 2).

**Table 2.** Performance of the Full Sample, Neutral, and Stratified Models in Predicting Foregoing Preventive Dental Care in the Extreme Gradient Boosting Model

Model	Standard threshold^a^					Group-specific threshold^b^			
	AUC (95% CI)	Sensitivity	Specificity	Precision^c^	F1^d^	Sensitivity	Specificity	Precision^c^	F1^d^
Full sample	0.84 (0.84-0.85)	0.86	0.68	0.82	0.84	0.78	0.77	0.85	0.81
Full sample applied to subgroups									
Race and ethnicity									
Asian	0.79 (0.75-0.83)	0.79	0.64	0.81	0.80	0.71	0.71	0.83	0.77
Black	0.78 (0.75-0.81)	0.94	0.40	0.84	0.89	0.76	0.66	0.88	0.81
Hispanic	0.81 (0.78-0.83)	0.93	0.41	0.85	0.89	0.74	0.74	0.91	0.82
White	0.84 (0.83-0.85)	0.79	0.78	0.81	0.80	0.76	0.80	0.82	0.79
Other^e^	0.80 (0.74-0.85)	0.89	0.52	0.83	0.86	0.73	0.69	0.86	0.79
Age, y									
18-64	0.82 (0.81-0.83)	0.86	0.63	0.82	0.84	0.75	0.76	0.86	0.80
≥65	0.88 (0.87-0.90)	0.86	0.79	0.83	0.85	0.83	0.83	0.85	0.84
Income									
High	0.83 (0.82-0.84)	0.80	0.74	0.79	0.80	0.76	0.78	0.81	0.78
Low	0.81 (0.79-0.83)	0.94	0.42	0.86	0.90	0.78	0.70	0.91	0.84
Neutral									
Race-neutral	0.84 (0.84-0.85)	0.86	0.68	0.82	0.84	0.77	0.77	0.86	0.81
Age-neutral	0.84 (0.84-0.85)	0.86	0.68	0.82	0.84	0.77	0.78	0.86	0.81
Income-neutral	0.84 (0.83-0.85)	0.86	0.68	0.82	0.84	0.78	0.77	0.85	0.81
Stratified									
Race and ethnicity									
Asian (n = 1935)	0.76 (0.73-0.81)	0.78	0.62	0.80	0.79	0.69	0.70	0.82	0.75
Black (n = 5138)	0.78 (0.75-0.81)	0.95	0.32	0.82	0.88	0.77	0.65	0.88	0.82
Hispanic (n = 7681)	0.80 (0.78-0.82)	0.94	0.37	0.84	0.89	0.72	0.74	0.91	0.80
White (n = 16 503)	0.85 (0.84-0.86)	0.80	0.77	0.80	0.80	0.77	0.80	0.81	0.79
Other (n = 977)^e^	0.76 (0.70-0.81)	0.87	0.46	0.81	0.84	0.69	0.69	0.85	0.76
Age, y									
18-64 (n = 25 052)	0.82 (0.81-0.83)	0.86	0.62	0.82	0.84	0.74	0.76	0.86	0.79
≥65 (n = 7182)	0.89 (0.87-0.90)	0.86	0.80	0.83	0.85	0.83	0.83	0.85	0.84
Income									
High (n = 20 751)	0.83 (0.82-0.84)	0.80	0.74	080	0.80	0.76	0.78	0.81	0.78
Low (n = 11 483)	0.80 (0.78-0.82)	0.95	0.38	0.86	0.90	0.76	0.71	0.91	0.83

Abbreviation: AUC, area under the receiver operating characteristic curve.

^a^ Standard threshold: 0.5.

^b^ Group-specific threshold was defined according to the prevalence of foregoing preventive dental care for each subgroup in the training data set (full sample, 0.66; White, 0.56; Black, 0.78; Hispanic, 0.79; Asian, 0.62; other or multiple racial groups, 0.72; age 18-64 years, 0.68; age ≥65 years, 0.60; high income, 0.58; and low income, 0.80).

^c^ Positive predictive value.

^d^ Harmonic mean of sensitivity and precision.

^e^ Other includes groups that were too small to be evaluated individually, such as American Indian and Alaska Native individuals, or those reporting multiple racial groups.

The results from the neutral models, generated without race, age, or income data, are presented in Table 2 and eTable 3 in Supplement 1. These models performed similarly to the full sample model for all evaluated metrics.

Results from the stratified models, generated specifically for each sociodemographic subsample, are reported in Table 2 and eTable 4 in Supplement 1. Overall, the predictive performance of the stratified models was similar to or lower than the full sample model, except among White adults (AUC, 0.85; 95% CI, 0.84-0.86). A lower performance was observed for Asian adults (AUC, 0.79; 95% CI, 0.75-0.83) when applying the full sample model, but the Asian subpopulation-specific model yielded an AUC of 0.76 (95% CI, 0.73-0.81).

**Figure 1.**
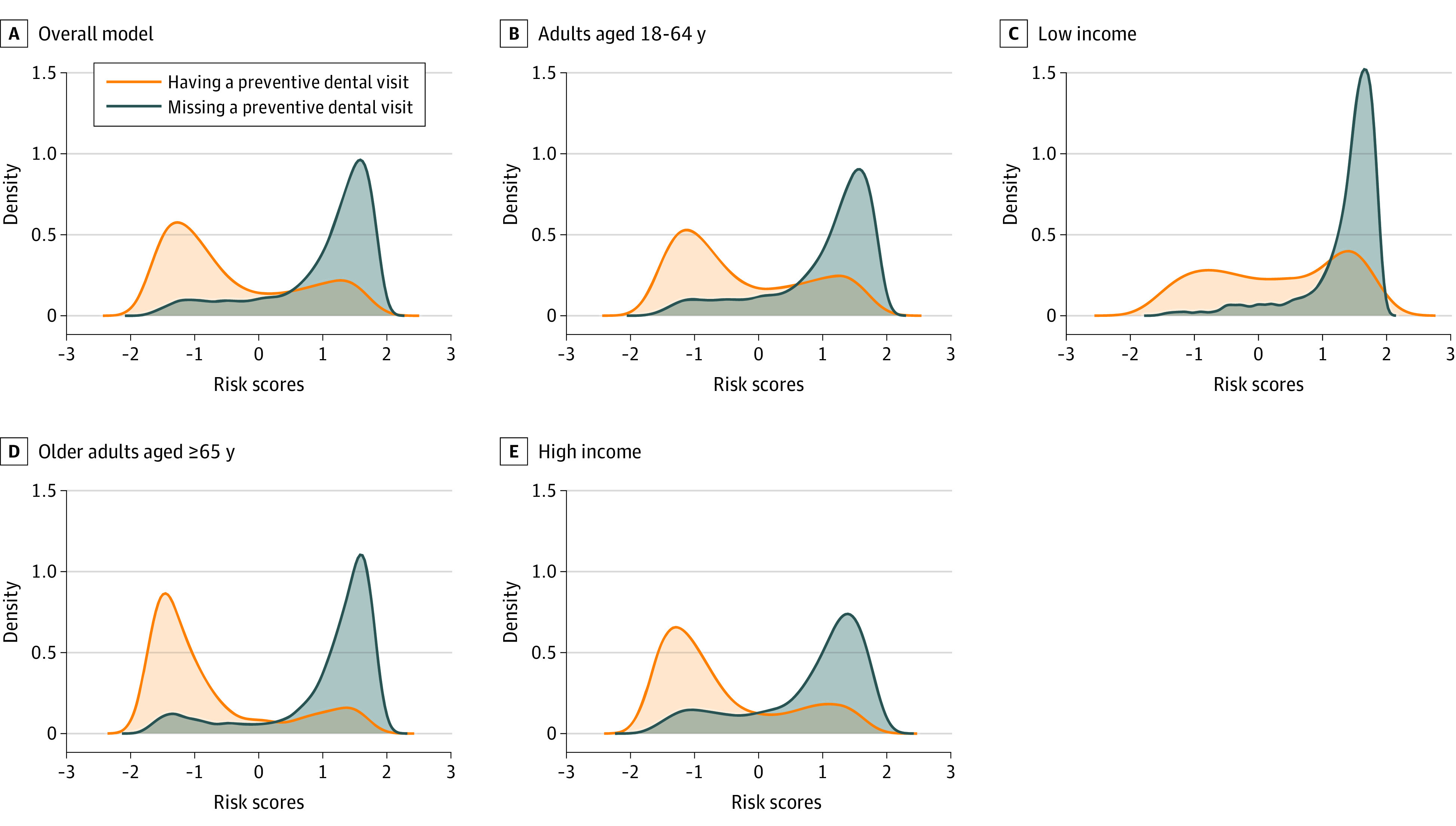
Density Plots for the Full Sample Model and for the Full Sample Model Applied to Age and Income Subgroups

**Figure 2.**
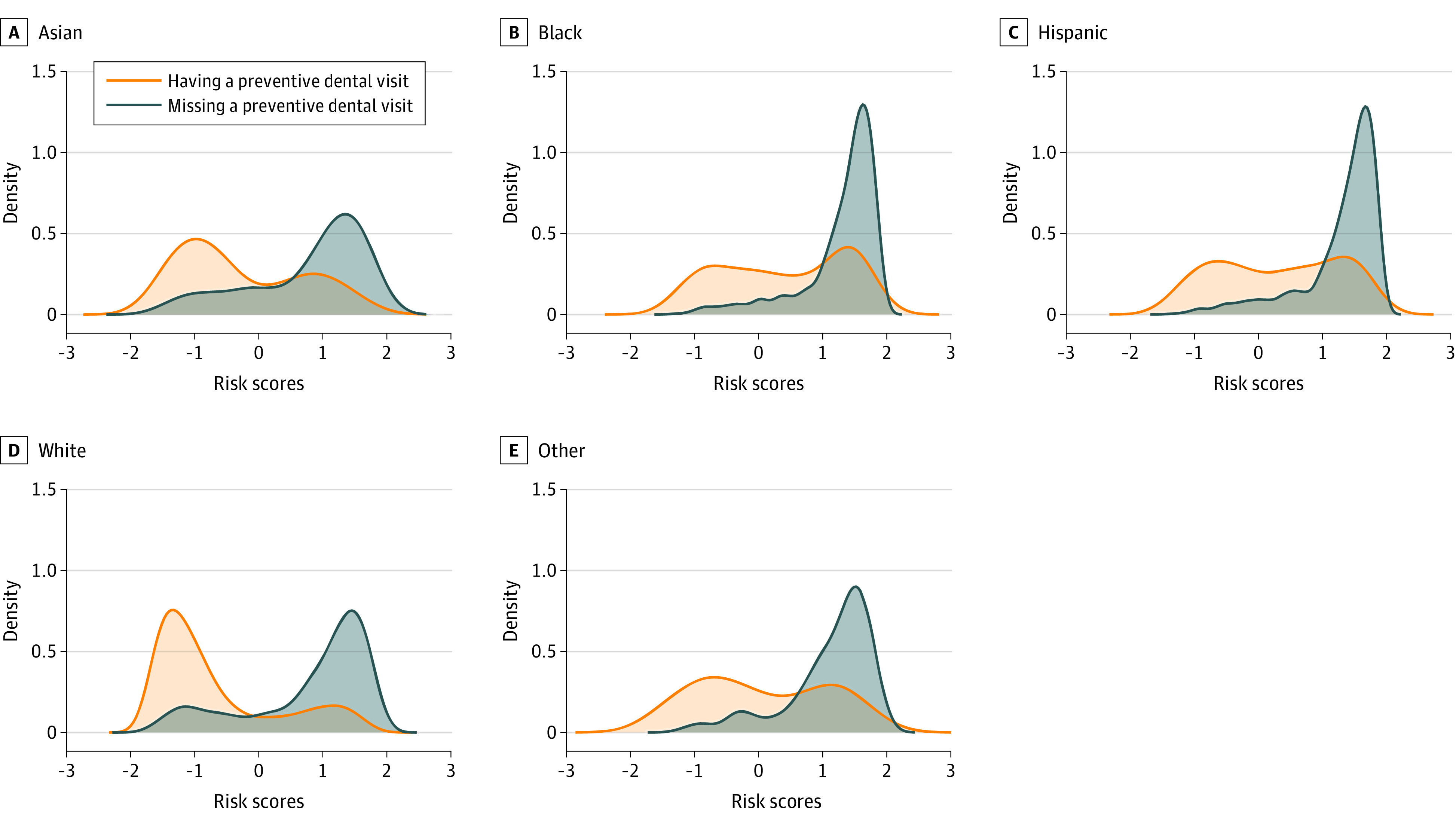
Density Plots for the Full Sample Model Applied to Race and Ethnicity Subgroups Other includes groups that were too small to be evaluated individually, such as American Indian and Alaska Native individuals, or those reporting multiple racial groups.

Figure 1 and Figure 2 show the prediction density of the full sample model, and the prediction density of the full sample model applied to each sociodemographic subgroup. The full sample model presents good discriminative ability, with low overlap between classes. Among subgroups, the best discriminative ability was observed among White adults, older adults, and high-income subpopulations, with good ability to identify both those who had and those who missed preventive dental care in the past year.

**Figure 3.**
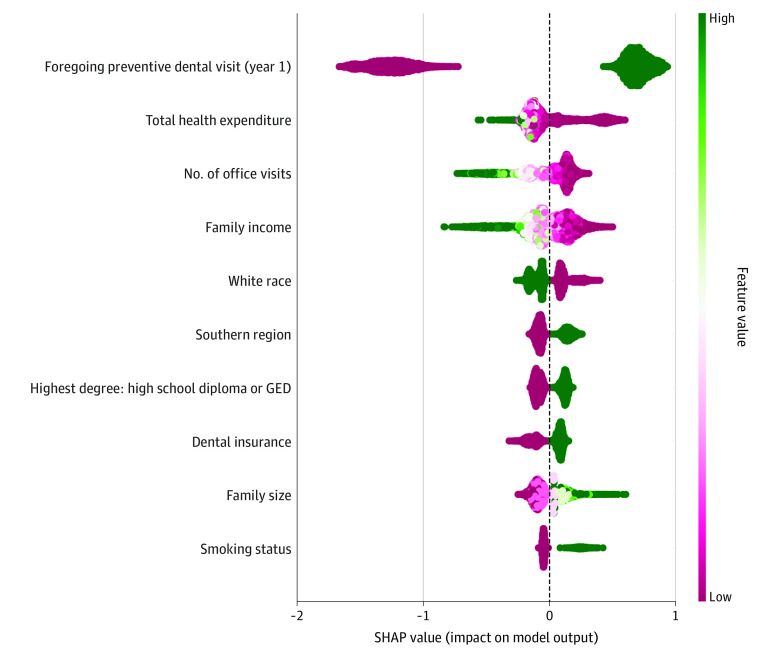
Main Contributors to Predicting Foregoing Preventive Dental Care Among Adults in the Study Sample, Measured by Shapley Additive Explanation (SHAP) Values The features are ordered according to their importance. The x-axis indicates the direction of the impact for each feature on the model output, with SHAP values higher than 0 indicating a positive impact. The feature value axis is colored according to the label of each predictor. For instance, foregoing preventive dental visit in the previous year was a dichotomous variable coded as 0 or 1, with 1 indicating foregone dental visit. Therefore, it is depicted in green, reflecting a higher value compared with 0. The SHAP value was positive for foregone dental visit, indicating that this predictor positively impacted the model. GED indicates general education development.

Figure 3 presents the density scatter plot of SHAP values, which represent the impact of each feature (predictor) on the model performance. The features in the figure are ordered according to their importance. The most important predictor was foregoing preventive dental visit in the past year, followed by features related to health care utilization, dental benefits, and sociodemographic characteristics.

The XGB algorithm had the highest performance. Findings from all other classifiers yielded consistent results (eTable 5 and eTable 4 in Supplement 1). The application of the SMOTE balancing technique did not improve our models’ performance (eTable 6 and eTable 7 in Supplement 1).

## Discussion

In this prognostic study, we predicted foregone preventive dental care among adults with high performance. However, our findings demonstrate that the ML algorithms were biased against certain sociodemographic groups, namely Asian, Black, Hispanic, and other race and ethnicity groups, individuals with low-income, and younger adults, who already experience significant challenges accessing preventive dental care.^7^ If these discriminatory models are put into practice, they could worsen an already unjust situation. Health outcomes and use of health care services are socially patterned; therefore, our findings regarding the algorithm’s unfairness in predicting foregoing preventive dental care may apply similarly to other health care outcomes.

ML algorithms have been criticized for their lack of transparency and interpretability,^10^ which can limit their acceptability to practitioners and patients. To encourage the adoption and use of artificial intelligence, it is important to identify and report the predictors that contribute most to the overall performance of the model. In our study, previous use of dental and health services, health expenditures, race and ethnicity, and income were the most significant factors, as expected; dental insurance was also ranked as an important predictor. Dental insurance is a strong determinant of use of dental services and poor oral health.^26^ In this analysis, we were not able to differentiate the source or the type of dental coverage, such as private or public, or level of coverage. Therefore, we stratified the sample according to family income level to capture the role of coverage for low-income adults, who are largely uninsured or covered by Medicaid.

Although ML is increasingly being used in health care,^27^ there are concerns about ethics, governance, regulations, and fairness. These issues must be considered throughout the development, validation, and implementation of ML models. One of the guiding principles recommended by the Expert Panel on Racial Bias and Healthcare Algorithms from the AHRQ and the National Institute on Minority Health and Health Disparities^28^ is that fairness of the model output across patient cohorts should be measured using distributive justice metrics, such as demographic parity and equalized odds. These measures assess whether the models generate unintentional discrimination due to an unequal impact on subgroups. Several measures have been proposed, focusing on different aspects of the prediction. For example, while demographic parity aims to match the proportion of positive predictions across subgroups, equalized odds intends to homogenize both true-positive and false-positive rates.^29^ However, there is currently a lack of standard methods for measuring and mitigating discrimination in ML models.^30,31,32^

When assessing fairness, it is important to consider race and ethnicity beyond binary comparisons between privileged and unprivileged groups. Comparisons between White and non-White or Black and White groups^33^ ignore historical and cultural characteristics of different racial and ethnic groups and can mask the performance of the models within individual groups. For example, our study found consistently poorer model performance for Black adults compared with other groups. Combining non-White individuals as 1 group would obscure this discrimination and hinder efforts to mitigate it. Similarly, the lack of data on Hispanic individuals, currently the largest racial and ethnic minority group in the US, would compromise the models’ fairness.^34^

Data bias resulting from a lack of high-quality data for minority racial and ethnic groups is another concern.^33^ Our study found that applying the full sample model to generate stratified models resulted in the largest reduction in performance among groups with smaller sample sizes, such as Asian individuals and those who identified as Other race and ethnicity or multiple racial or ethnic groups. These data biases may reinforce inequities, especially when the algorithms are informed by data from health care encounters to which vulnerable groups have limited access.^33^

Our findings demonstrated that generating a stratified model does not improve model performance for the underrepresented groups and can even increase model bias. To address model fairness, there are 2 recommended approaches: developing a group-blinding classifier (fairness through unawareness*,* neutral-models*)* or splitting classifiers (fairness through awareness, stratified models).^35^ The group-blinding classifier generates models without sensitive features, such as race and ethnicity, or income. However, this approach does not capture unfair inherent biases, as predictors can be proxies for the protected information.^36^ In this study, we examined both approaches, generating neutral models and models stratified for each group, but both approaches were ineffective in addressing model unfairness in our study.

Scholars have proposed various bias mitigation approaches at different stages of the ML development pipeline,^31,37,38,39^ including preprocessing techniques, like resampling; in-processing, such as constraints and regularization; and postprocessing methods like a group-specific modification or decision thresholds.^31,39^ In our study, we established target thresholds for each subgroup^30^ and adopted the group-specific prevalence threshold, but this was deemed unsuitable, since a model that is more sensitive than specific is preferable in predicting missed preventive health care opportunities.

### Limitations

This study has some limitations. The scientific community currently lacks^40^ consensus on how to best address survey weights in prediction modeling. We found that our survey-weighted and unweighted samples were similar, so we did not incorporate MEPS survey weights in our algorithms. Additionally, the MEPS is based on self-reported data, which are susceptible to recall bias. Moreover, the use of only 1 data set limits our ability to generalize models’ performance in different settings.

## Conclusions

In this prognostic study using cohort data, our findings suggest that ML models could accurately predict some adults at risk of foregoing preventive dental care. However, our findings also indicate that our ML models had a lower performance for non-White, low-income, and younger adults. Future research will benefit from standard benchmarks for fairness evaluation, mitigation, and reporting. These results can inform targeted prevention efforts to reduce disparities in oral health and underscore the importance of evaluating models during their development and testing to avoid exacerbating existing biases.

## Notes

Supplement 1. **eTable 1.** Variable Definitions **eTable 2.** Characteristics of Training and Testing Samples **eFigure 1.** Ethnic and Racial Distribution of the Sample, Unweighted and Survey-Weighted Proportions **eFigure 2.** Foregoing Preventive Dental Care by Ethnic and Racial Groups **eTable 3.** Performance of the Neutral Models in Predicting Foregoing Preventive Dental Care **eTable 4.** Performance of the Group-Specific Models in Predicting Foregoing Preventive Dental Care **eTable 5.** Performance of the Overall Model Predicting Foregoing Preventive Dental Care **eTable 6.** Performance of the Overall Model Predicting Foregoing Preventive Dental Care, Applying the Synthetic Minority Over-Sampling Technique (SMOTE) in the Training Dataset **eTable 7.** Performance of the Group-Specific Models in Predicting Foregoing Preventive Dental Care, Applying the Synthetic Minority Over-Sampling Technique (SMOTE) in the Training Dataset

Supplement 2.
Data Sharing Statement
