# Supplementary material for: Fairness of Machine Learning Algorithms for Predicting Foregone Preventive Dental Care for Adults
Source: JAMA Netw Open. 2023 Nov 3;6(11):e2341625. doi: 10.1001/jamanetworkopen.2023.41625 (PMC10625037; doi:10.1001/jamanetworkopen.2023.41625)
Supplement: Supplement 1. — eTable 1. Variable Definitions eTable 2. Characteristics of Training and Testing Samples eFigure 1. Ethnic and Racial Distribution of the Sample, Unweighted and Survey-Weighted Proportions eFigure 2. Foregoing Preventive Dental Care by Ethnic and Racial Groups eTable 3. Performance of the Neutral Models in Predicting Foregoing Preventive Dental Care eTable 4. Performance of the Group-Specific Models in Predicting Foregoing Preventive Dental Care eTable 5. Performance of the Overall Model Predicting Foregoing Preventive Dental Care eTable 6. Performance of the Overall Model Predicting Foregoing Preventive Dental Care, Applying the Synthetic Minority Over-Sampling Technique (SMOTE) in the Training Dataset eTable 7. Performance of the Group-Specific Models in Predicting Foregoing Preventive Dental Care, Applying the Synthetic Minority Over-Sampling Technique (SMOTE) in the Training Dataset [file jamanetwopen-e2341625-s001.pdf]

Supplemental Online Content

Schuch HS, Furtado M, Silva GFS, Kawachi I, Filho APC, Elani HW. Fairness of machine learning algorithms for predicting foregone preventive dental care for adults. *JAMA Netw Open*. 2023;6(11):e2341625. doi:10.1001/jamanetworkopen.2023.41625

eTable 1. Characteristics of Training and Testing Samples

eFigure 1. Ethnic and Racial Distribution of the Sample, Unweighted and Survey-Weighted Proportions

eFigure 2. Foregoing Preventive Dental Care by Ethnic and Racial Groups

eTable 2. Performance of the Overall Model Predicting Foregoing Preventive Dental Care

eTable 3. Performance of the Group-Specific Models in Predicting Foregoing Preventive Dental Care

eTable 4. Performance of the Overall Model Predicting Foregoing Preventive Dental Care, Applying the Synthetic Minority Over-Sampling Technique (SMOTE) in the Training Dataset

eTable 5. Performance of the Group-Specific Models in Predicting Foregoing Preventive Dental Care, Applying the Synthetic Minority Over-Sampling Technique (SMOTE) in the Training Datasets

eTable 6. Performance of the Neutral Models in Predicting Foregoing Preventive Dental Care

eTable 7. Variable Definitions

This supplemental material has been provided by the authors to give readers additional information about their work.

© 2023 Schuch HS et al. *JAMA Network Open.*

eTable 1. Variables' definitions.

Variable (MEPS name)	Definition/categories	Variable (MEPS name)	Definition/categories
**Age** (agey1x)	Continuous age in years	**Sex** (sex)	Male; Female
**Race** (racethx)	Hispanic; Non-Hispanic, Asian; Non-Hispanic, Black; Non-Hispanic, white; Non-Hispanic, other or multiple race.	**Education** – highest degree (hideg)	Some college/College graduate; High school diploma/GED; Less than high school
**Occupation** (occct1h)	Manag., business, and financial oper; Professi. and related occ; Service occ; Sales and related occ; Office and admin. support; Farming, fishing, and forestry; Construct., extract., and maint.; Producti., transp., mtrl moving; Military occ; Unclassified.	**Family income in relation to poverty line** (povcaty1)	High income (400+%); Middle income (200-399%); Low income (125-199%); Nearly poor (100-124%); Poor/negative (<100%)
**Family income** (famincy1)	Continuous annual family earnings in $	**Family size** (famszey1)	Number of people in the household.
**Marital status** (marryy1x)	Married; Not married	**Employment status** (empst1h)	Employed; Not employed
**Census region** (regiony1)	Northeast; Midwest; South; West	**Country of birth** (bornusa)	USA; Other country.
**Language of SAQ interview** (adlang2)	English; Spanish.	**Retirement status** (evretiy1)	No; Yes
**Heart disease** (chddxy1; angidxy1; midxy1; ohrthdxy1)	No; Yes	**Hypertension** (hibpdxy1)	No; Yes
**Medical insurance** (inscovy1)	Any private; Public only; Uninsured	**Dental insurance** (dntins1)	Yes; No
**High cholesterol** (choldxy1)	No; Yes	**Emphysema** (emphdxy1)	No; Yes
**Bronchitis** (chbron1)	No; Yes	**Diabetes** (diabdxy1)	No; Yes
**Cancer** (cancery1)	No; Yes	**Asthma** (asthdxy1)	No; Yes
**Arthritis** (arthdxy1)	No; Yes	**Stroke** (strkdxy1)	No; Yes
**Perceived health status** (rthlth1)	Excellent; Very good; Good; Fair; Poor	**Perceived health status 2** (adgenh2)	Excellent; Very good; Good; Fair; Poor
**Perceived mental health status** (mnhlth1)	Excellent; Very good; Good; Fair; Poor	**Feeling hopeless** (adhope2)	None of the time; Little of the time; Some of the time; Most of the time; All the time
**Feeling down or depressed** (addprs2)	Not at all; Several days; More than half the days; Nearly every day; All the time	**Little interest in things** (adintr2)	Not at all; Several days; More than half the days; Nearly every day; All the time
**Cognitive limitations** (coglim1)	No; Yes	**Physical limitations** (wlklim1)	No; Yes
**Any limitations** (actlim1)	No; Yes	**Health limitations** (addaya2)	No; Yes
**Routine medical care** (adrtcr2 - panels 21 and 22, adrtcr4 - panel 23)	Yes, No	**# Office-based visits** (obtotvy1)	Continuous number of office visits in the first year
**# Emergency room visits** (ertoty1)	Continuous number of ER visits	**# Prescribed medications** (rxtoty1)	Continuous number of prescribed medications
**Total healthcare expenditure** (dvtoty1)	Continuous amount expended with healthcare	**Total ER expenditure** (totexpy1)	Continuous amount expended with emergency room visit
**Total ER Medicaid expenditure** (erfmcry1)	Continuous amount expended with ED covered by Medicaid	**Total ER Medicare expenditure** (erfexpy1)	Continuous amount expended with ED covered by Medicare
**Physical activity** (phyexe3)	Yes, No	**BMI** (21- bmindx3, 22-adbmi4, 23-adbmi2)	Underweight; Healthy; Overweight/obese

© 2023 Schuch HS et al. *JAMA Network Open.*

**Smoking** (oftsmk5 and adsmok2 (panel 21) oftsmk3 (panel 22) oftsmk3 and adsmok4 (panel 23)	No; Yes	**Restorative dental visit** (filling, rootcanl, bridges, crowns, inlay, implant, dentures, repair (panel 21); filling, rootcanl, bridges, implant (panel 22); filling_m18, rootcanl_m18, bridges_m18, implant_m18 (panel 23))	No; Yes
**Emergency dental visit** (abscess, extract, oralsurg (panel 21) oralsurg (panel 22); oralsurg_m18 (panel 23))	No; Yes	**Preventive dental visit** (examine, clenteth, fluoride, sealant, denthyg, reclvis (panel 21); examine, clenteth, fluoride, sealant, denthyg (panels 22 and 23))	Yes, No

© 2023 Schuch HS et al. *JAMA Network Open.*

eTable 2. Characteristics of training and testing samples.

	2016-18 (training)		2018-19 (test)	
	N = 21,787		N = 10,447	
**Gender**	n	%	n	%
Male	10,009	45.9	4,839	46.3
Female	11,778	54.1	5,608	53.7
**Age, y**				
Mean, ± SD	47.9 (18.1)		49.7 (18.5)	
18-64	17,241	79.1	7,811	74.8
65-85	4,546	20.9	2,636	25.2
**Education**				
Less than high school	4,625	21.4	1,908	18.4
High school diploma or GED	9,303	43.1	4,397	42.5
Some college or college graduate	7,662	35.5	4,053	39.1
**Race and ethnicity**				
Asian	1,384	6.3	551	5.3
Black	3,627	16.6	1,511	14.5
Hispanic	5,524	25.4	2,157	20.6
White	10,601	48.7	5,902	56.5
Other[a]	651	3.0	326	3.1
**Family income, % of Federal Poverty Line**				
<200	8,088	37.1	3,395	32.5
200-399	6,327	29.0	3,025	29.0
≥400	7,372	33.8	4,027	38.5
**Heath insurance**				
Uninsured	2,440	11.2	859	8.2
Public only	6,080	27.9	3,010	28.8
Any private	13,267	60.9	6,578	63.0
**Dental insurance**				
No	13,666	62.8	6,341	60.8
Yes	8,083	37.2	4,093	39.2
**Smoking**	3,916	18.0	2,358	22.6
**Medical condition**				
Asthma	2,579	11.8	1,387	13.3
Arthritis	5,670	26.0	2,823	27.0
Bronchitis	481	2.3	256	2.5
Cancer	2,091	9.6	1,272	12.2
Diabetes	2,670	12.3	1,346	12.9
Emphysema	472	2.2	213	2.0
Heart diseases	3,087	14.2	1,601	15.3
Hypertension	7,517	34.5	3,698	35.4
High cholesterol levels	6,629	30.5	3,365	32.2
Stroke	927	4.3	485	4.6

Medical Expenditure Panel Survey, 2016-2019. [a]nIncludes groups that were too small to be evaluated individually, such as American Indians and Alaska Natives, or those reporting multiple racial groups.

© 2023 Schuch HS et al. *JAMA Network Open.*

eFigure 1. Ethnic and racial distribution of the sample, unweighted and survey-weighted proportions.

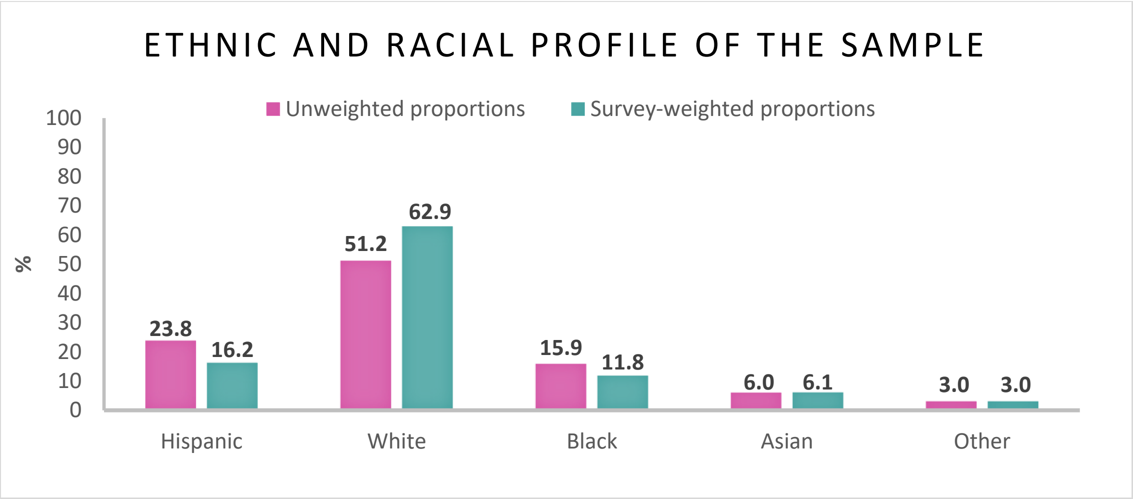

Note: “Other” includes groups that were too small to be evaluated individually, such as American Indians and Alaska Natives, or those reporting multiple racial groups.

eFigure 2. Foregoing preventive dental care by ethnic and racial groups. Unweighted and survey-weighted proportions.

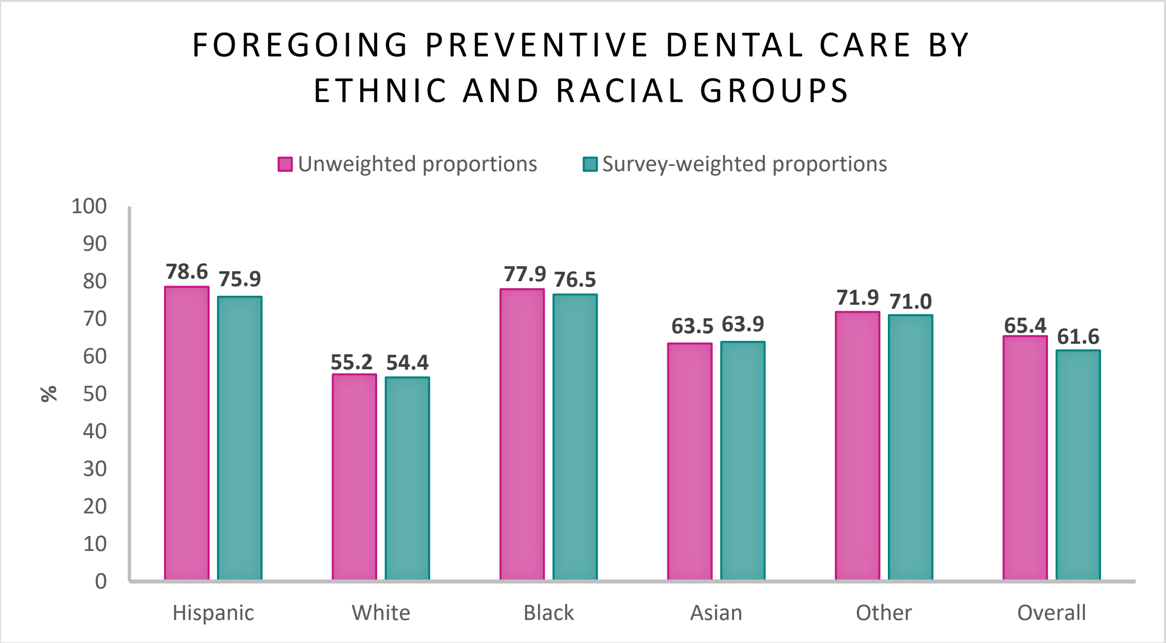

Note: “Other” includes groups that were too small to be evaluated individually, such as American Indians and Alaska Natives, or those reporting multiple racial groups.

© 2023 Schuch HS et al. *JAMA Network Open.*

eTable 3. Performance of the neutral models in predicting foregoing preventive dental care.

	Standard threshold[a]					Group-specific threshold[b]			
	**AUROC (95% CI)**	**Sensitivity**	**Specificity**	**Precision[c]**	**F1[d]**	**Sensitivity**	**Specificity**	**Precision[c]**	**F1[d]**
**Light Gradient Boosting**									
Race-neutral	0.84 (0.83-0.85)	0.85	0.68	0.82	0.84	0.76	0.78	0.86	0.81
Age-neutral	0.84 (0.83-0.85)	0.85	0.69	0.82	0.84	0.76	0.78	0.85	0.81
Income-neutral	0.84 (0.83-0.85)	0.85	0.69	0.82	0.84	0.77	0.77	0.86	0.81
**Random Forest**									
Race-neutral	0.84 (0.83-0.85)	0.86	0.67	0.82	0.84	0.74	0.80	0.86	0.80
Age-neutral	0.84 (0.83-0.85)	0.87	0.66	0.82	0.84	0.74	0.80	0.86	0.80
Income-neutral	0.84 (0.83-0.85)	0.87	0.66	0.81	0.84	0.75	0.79	0.86	0.80
**Catboost**									
Race-neutral	0.84 (0.83-0.84)	0.85	0.68	0.82	0.84	0.76	0.77	0.85	0.80
Age-neutral	0.84 (0.83-0.84)	0.86	0.67	0.82	0.84	0.77	0.77	0.85	0.81
Income-neutral	0.83 (0.83-0.84)	0.86	0.67	0.82	0.84	0.76	0.77	0.85	0.80
**SMOTE**									
**Extreme Gradient Boosting**									
Race-neutral	0.83 (0.82-0.84)	0.84	0.68	0.82	0.83	0.75	0.77	0.85	0.80
Age-neutral	0.83 (0.82-0.84)	0.84	0.69	0.82	0.83	0.75	0.77	0.85	0.79
Income-neutral	0.83 (0.82-0.83)	0.85	0.67	0.82	0.83	0.75	0.77	0.85	0.79
**Light Gradient Boosting**									
Race-neutral	0.84 (0.83-0.85)	0.83	0.70	0.83	0.83	0.74	0.79	0.86	0.79
Age-neutral	0.84 (0.83-0.85)	0.83	0.70	0.83	0.83	0.74	0.79	0.86	0.79
Income-neutral	0.84 (0.83-0.85)	0.84	0.69	0.82	0.83	0.74	0.79	0.86	0.79
**Random Forest**									
Race-neutral	0.84 (0.83-0.85)	0.82	0.71	0.83	0.83	0.65	0.84	0.88	0.75
Age-neutral	0.84 (0.83-0.85)	0.82	0.71	0.83	0.83	0.66	0.84	0.88	0.75
Income-neutral	0.84 (0.83-0.85)	0.82	0.71	0.83	0.83	0.66	0.84	0.88	0.75
**Catboost**									
Race-neutral	0.83 (0.82-0.84)	0.84	0.69	0.82	0.83	0.74	0.78	0.86	0.79
Age-neutral	0.83 (0.83-0.84)	0.84	0.70	0.83	0.83	0.74	0.79	0.86	0.80
Income-neutral	0.83 (0.83-0.84)	0.84	0.69	0.82	0.83	0.73	0.78	0.85	0.79

[a]Standard threshold: 0.5. [b]Group-specific threshold was defined according to the prevalence of foregoing preventive dental care for each subgroup in the training dataset. For the full sample: 0.66; White: 0.56; Black: 0.78; Hispanic: 0.79; Asian: 0.62; Other or multiple races: 0.72; 18-64: 0.68; 65+: 0.60; high income: 0.58; and low income: 0.80. [c]Precision: Positive predictive value. [d]F1 score: harmonic mean of sensitivity and precision. [e]"Other" includes groups that were too small to be evaluated individually, such as American Indians and Alaska Natives, or those reporting multiple racial groups.

© 2023 Schuch HS et al. *JAMA Network Open.*

eTable 4. Performance of the subgroup-stratified models in predicting foregoing preventive dental care.

	Standard threshold[a]					Group-specific threshold[b]			
	**AUROC (95% CI)**	**Sensitivity**	**Specificity**	**Precision[c]**	**F1[d]**	**Sensitivity**	**Specificity**	**Precision[c]**	**F1[d]**
**Light Gradient Boosting**									
Asian	0.76 (0.72-0.80)	0.75	0.64	0.80	0.77	0.66	0.76	0.84	0.74
Black	0.77 (0.74-0.80)	0.94	0.33	0.82	0.88	0.74	0.65	0.88	0.80
Hispanic	0.80 (0.77-0.82)	0.94	0.37	0.84	0.89	0.70	0.75	0.91	0.79
White	0.84 (0.83-0.85)	0.80	0.77	0.80	0.80	0.76	0.80	0.81	0.79
Other[e]	0.74 (0.68-0.80)	0.86	0.44	0.80	0.83	0.66	0.69	0.85	0.74
18-64	0.82 (0.81-0.83)	0.85	0.63	0.82	0.84	0.73	0.76	0.86	0.79
≥65	0.88 (0.86-0.89)	0.86	0.80	0.83	0.84	0.82	0.83	0.85	0.83
High income	0.83 (0.82-0.84)	0.80	0.74	0.79	0.79	0.75	0.77	0.81	0.78
Low income	0.80 (0.78-0.82)	0.94	0.39	0.86	0.90	0.76	0.70	0.91	0.83
**Random Forest**									
Asian	0.78 (0.75-0.82)	0.81	0.61	0.81	0.81	0.66	0.77	0.85	0.74
Black	0.77 (0.75-0.80)	0.96	0.24	0.81	0.88	0.67	0.73	0.89	0.76
Hispanic	0.80 (0.77-0.82)	0.96	0.29	0.82	0.88	0.64	0.79	0.91	0.76
White	0.85 (0.84-0.86)	0.80	0.77	0.80	0.80	0.76	0.81	0.82	0.79
Other[e]	0.77 (0.71-0.83)	0.93	0.33	0.78	0.85	0.61	0.74	0.86	0.72
18-64	0.82 (0.81-0.83)	0.88	0.59	0.81	0.84	0.70	0.79	0.87	0.78
≥65	0.89 (0.87-0.90)	0.86	0.79	0.82	0.84	0.81	0.84	0.86	0.83
High income	0.83 (0.82-0.84)	0.82	0.72	0.79	0.80	0.73	0.80	0.82	0.77
Low income	0.80 (0.78-0.82)	0.97	0.29	0.84	0.90	0.68	0.92	0.77	0.79
**Catboost**									
Asian	0.69 (0.64-0.74)	0.71	0.67	0.81	0.76	0.79	0.46	0.82	0.59
Black	0.72 (0.68-0.75)	0.91	0.33	0.82	0.86	0.74	0.60	0.86	0.80
Hispanic	0.78 (0.76-0.80)	0.91	0.42	0.84	0.88	0.73	0.72	0.90	0.81
White	0.84 (0.82-0.85)	0.79	0.76	0.79	0.79	0.75	0.79	0.80	0.78
Other[e]	0.66 (0.59-0.73)	0.78	0.38	0.77	0.77	0.67	0.59	0.81	0.73
18-64	0.81 (0.80-0.82)	0.85	0.62	0.81	0.83	0.73	0.76	0.86	0.79
≥65	0.87 (0.85-0.88)	0.84	0.79	0.82	0.83	0.80	0.82	0.84	0.82
High income	0.82 (0.81-0.83)	0.79	0.73	0.78	0.78	0.74	0.78	0.80	0.77
Low income	0.78 (0.76-0.80)	0.92	0.42	0.86	0.89	0.75	0.70	0.91	0.82

[a]Standard threshold: 0.5. [b]Group-specific threshold was defined according to the prevalence of foregoing preventive dental care for each subgroup in the training dataset. For the full sample: 0.66; White: 0.56; Black: 0.78; Hispanic: 0.79; Asian: 0.62; Other or multiple races: 0.72; 18-64: 0.68; 65+: 0.60; high income: 0.58; and low income: 0.80. [c]Precision: Positive predictive value. [d]F1 score: harmonic mean of sensitivity and precision. [e]"Other" includes groups that were too small to be evaluated individually, such as American Indians and Alaska Natives, or those reporting multiple racial groups.

© 2023 Schuch HS et al. *JAMA Network Open.*

eTable 5. Performance of the overall model predicting foregoing preventive dental care.

	Standard threshold[a]					Group-specific threshold[b]			
	**AUROC (95% CI)**	**Sensitivity**	**Specificity**	**Precision[c]**	**F1[d]**	**Sensitivity**	**Specificity**	**Precision[c]**	**F1[d]**
**Light Gradient Boosting**									
Overall Model	0.84 (0.83-0.85)	0.85	0.68	0.82	0.84	0.77	0.78	0.86	0.81
Asian	0.79 (0.75-0.83)	0.78	0.64	0.81	0.80	0.70	0.72	0.83	0.76
Black	0.78 (0.75-0.81)	0.93	0.38	0.83	0.88	0.76	0.66	0.88	0.82
Hispanic	0.80 (0.78-0.82)	0.93	0.41	0.84	0.89	0.74	0.74	0.91	0.81
White	0.84 (0.83-0.85)	0.79	0.78	0.80	0.80	0.76	0.80	0.82	0.79
Other[e]	0.81 (0.76-0.86)	0.91	0.47	0.82	0.86	0.76	0.74	0.89	0.82
18-64	0.82 (0.81-0.83)	0.85	0.63	0.82	0.83	0.74	0.77	0.86	0.80
≥65	0.89 (0.87-0.90)	0.86	0.80	0.83	0.84	0.83	0.83	0.85	0.84
High income	0.83 (0.82-0.84)	0.79	0.74	0.79	0.79	0.74	0.78	0.81	0.77
Low income	0.81 (0.79-0.83)	0.95	0.40	0.86	0.90	0.78	0.70	0.91	0.84
**Random Forest**									
Overall Model	0.84 (0.83-0.85)	0.87	0.66	0.81	0.84	0.74	0.80	0.86	0.80
Asian	0.79 (0.76-0.83)	0.83	0.60	0.81	0.82	0.72	0.71	0.83	0.77
Black	0.78 (0.76-0.81)	0.95	0.34	0.83	0.88	0.67	0.73	0.89	0.77
Hispanic	0.81 (0.79-0.83)	0.94	0.37	0.84	0.89	0.67	0.79	0.92	0.77
White	0.84 (0.83-0.85)	0.81	0.76	0.81	0.80	0.77	0.80	0.81	0.79
Other[e]	0.80 (0.74-0.86)	0.91	0.53	0.84	0.87	0.71	0.76	0.88	0.79
18-64	0.82 (0.81-0.83)	0.88	0.60	0.81	0.84	0.70	0.79	0.87	0.78
≥65	0.89 (0.87-0.90)	0.87	0.78	0.82	0.84	0.82	0.84	0.85	0.84
High income	0.83 (0.82-0.84)	0.82	0.73	0.79	0.80	0.76	0.78	0.81	0.78
Low income	0.81 (0.79-0.83)	0.96	0.34	0.85	0.90	0.68	0.77	0.92	0.78
**Catboost**									
Overall Model	0.84 (0.84-0.85)	0.86	0.68	0.82	0.84	0.78	0.77	0.85	0.81
Asian	0.79 (0.70-0.78)	0.80	0.63	0.81	0.80	0.73	0.70	0.83	0.77
Black	0.78 (0.75-0.81)	0.93	0.42	0.84	0.88	0.77	0.65	0.88	0.82
Hispanic	0.80 (0.78-0.83)	0.93	0.43	0.85	0.89	0.76	0.73	0.91	0.82
White	0.85 (0.84-0.86)	0.80	0.77	0.80	0.80	0.77	0.79	0.81	0.79
Other[e]	0.78 (0.72-0.84)	0.91	0.56	0.84	0.88	0.75	0.67	0.85	0.80
18-64	0.82 (0.81-0.83)	0.86	0.63	0.82	0.84	0.75	0.75	0.86	0.80
≥65	0.88 (0.87-0.90)	0.86	0.80	0.83	0.84	0.84	0.82	0.84	0.84
High income	0.83 (0.82-0.84)	0.80	0.74	0.79	0.80	0.76	0.77	0.81	0.78
Low income	0.80 (0.78-0.82)	0.94	0.42	0.86	0.90	0.80	0.69	0.91	0.85

[a]Standard threshold: 0.5. [b]Group-specific threshold was defined according to the prevalence of foregoing preventive dental care for each subgroup in the training dataset. For the full sample: 0.66; White: 0.56; Black: 0.78; Hispanic: 0.79; Asian: 0.62; Other or multiple races: 0.72; 18-64: 0.68; 65+: 0.60; high income: 0.58; and low income: 0.80. [c]Precision: Positive predictive value. [d]F1 score: harmonic mean of sensitivity and precision. [e]"Other" includes groups that were too small to be evaluated individually, such as American Indians and Alaska Natives, or those reporting multiple racial groups.

© 2023 Schuch HS et al. *JAMA Network Open.*

eTable 6. Performance of the overall model predicting foregoing preventive dental care, applying SMOTE.

	Standard threshold[a]					Group-specific threshold[b]			
	AUROC (95% CI)	Sensitivity	Specificity	Precision[c]	F1[d]	Sensitivity	Specificity	Precision[c]	F1[d]
**Extreme Gradient Boosting**									
Overall Model	0.83 (0.82-0.84)	0.84	0.68	0.82	0.83	0.75	0.77	0.85	0.80
Asian	0.76 (0.72-0.81)	0.76	0.65	0.81	0.78	0.71	0.70	0.82	0.76
Black	0.77 (0.74-0.80)	0.94	0.40	0.84	0.89	0.77	0.63	0.87	0.82
Hispanic	0.79 (0.76-0.81)	0.91	0.48	0.86	0.88	0.71	0.74	0.90	0.79
White	0.83 (0.82-0.84)	0.78	0.76	0.79	0.79	0.75	0.79	0.81	0.77
Other[e]	0.73 (0.67-0.79)	0.90	0.46	0.81	0.85	0.69	0.67	0.84	0.76
18-64	0.80 (0.79-0.81)	0.84	0.63	0.82	0.83	0.72	0.74	0.85	0.78
≥65	0.88 (0.86-0.89)	0.86	0.80	0.83	0.84	0.82	0.82	0.85	0.83
High income	0.82 (0.81-0.83)	0.78	0.74	0.79	0.79	0.73	0.78	0.81	0.77
Low income	0.78 (0.76-0.80)	0.94	0.43	0.86	0.90	0.73	0.78	0.81	0.77
**Light Gradient Boosting**									
Overall Model	0.84 (0.83-0.85)	0.84	0.70	0.83	0.83	0.74	0.79	0.86	0.79
Asian	0.78 (0.74-0.82)	0.77	0.65	0.81	0.79	0.69	0.73	0.84	0.76
Black	0.78 (0.75-0.81)	0.92	0.42	0.84	0.88	0.74	0.66	0.88	0.80
Hispanic	0.81 (0.78-0.83)	0.90	0.48	0.86	0.88	0.70	0.75	0.91	0.79
White	0.84 (0.83-0.85)	0.78	0.79	0.81	0.79	0.74	0.81	0.82	0.78
Other[e]	0.79 (0.73-0.84)	0.89	0.57	0.84	0.86	0.72	0.73	0.88	0.79
18-64	0.81 (0.80-0.83)	0.83	0.65	0.83	0.82	0.71	0.78	0.86	0.78
≥65	0.88 (0.87-0.90)	0.84	0.81	0.84	0.84	0.81	0.84	0.86	0.83
High income	0.83 (0.82-0.84)	0.77	0.76	0.80	0.79	0.72	0.80	0.82	0.77
Low income	0.80 (0.78-0.82)	0.93	0.44	0.87	0.90	0.74	0.71	0.91	0.81
**Random Forest**									
Overall Model	0.84 (0.83-0.85)	0.82	0.71	0.83	0.83	0.66	0.84	0.88	0.75
Asian	0.78 (0.75-0.82)	0.74	0.68	0.82	0.78	0.61	0.83	0.88	0.72
Black	0.78 (0.75-0.81)	0.92	0.41	0.84	0.88	0.52	0.82	0.91	0.66
Hispanic	0.81 (0.78-0.83)	0.89	0.49	0.86	0.87	0.51	0.89	0.94	0.66
White	0.84 (0.83-0.85)	0.76	0.80	0.82	0.79	0.70	0.84	0.84	0.76
Other[e]	0.79 (0.73-0.84)	0.88	0.57	0.84	0.86	0.54	0.84	0.90	0.68
18-64	0.80 (0.79-0.81)	0.84	0.62	0.82	0.83	0.73	0.75	0.85	0.78
≥65	0.88 (0.87-0.89)	0.85	0.80	0.83	0.84	0.81	0.83	0.85	0.83
High income	0.82 (0.81-0.83)	0.78	0.73	0.79	0.78	0.73	0.77	0.80	0.76
Low income	0.79 (0.77-0.81)	0.93	0.43	0.86	0.90	0.76	0.70	0.91	0.83
**Catboost**									
Overall Model	0.83 (0.82-0.84)	0.84	0.70	0.83	0.83	0.74	0.79	0.86	0.79
Asian	0.77 (0.73-0.81)	0.77	0.65	0.82	0.79	0.69	0.71	0.83	0.76
Black	0.77 (0.74-0.80)	0.92	0.41	0.84	0.88	0.75	0.68	0.89	0.81
Hispanic	0.80 (0.77-0.82)	0.90	0.52	0.87	0.88	0.68	0.77	0.91	0.78
White	0.84 (0.83-0.85)	0.77	0.78	0.81	0.79	0.73	0.81	0.82	0.77
Other[e]	0.78 (0.72-0.83)	0.91	0.46	0.81	0.86	0.79	0.63	0.85	0.82
18-64	0.81 (0.80-0.82)	0.83	0.65	0.83	0.83	0.71	0.77	0.86	0.78
≥65	0.88 (0.87-0.89)	0.84	0.81	0.84	0.84	0.80	0.84	0.85	0.83
High income	0.82 (0.81-0.83)	0.78	0.75	0.80	0.79	0.72	0.79	0.81	0.76
Low income	0.79 (0.77-0.81)	0.92	0.47	0.87	0.90	0.73	0.72	0.91	0.81

[a]Standard threshold: 0.5. [b]Group-specific threshold was defined according to the prevalence of foregoing preventive dental care for each subgroup in the training dataset. For the full sample: 0.66; White: 0.56; Black: 0.78; Hispanic: 0.79; Asian: 0.62; Other or multiple races: 0.72; 18-64: 0.68; 65+: 0.60; high income: 0.58; and low income: 0.80. [c]Precision: Positive predictive value. [d]F1 score: harmonic mean of sensitivity and precision. [e]"Other" includes groups that were too small to be evaluated individually, such as American Indians and Alaska Natives, or those reporting multiple racial groups.

© 2023 Schuch HS et al. *JAMA Network Open.*

eTable 7. Performance of the stratified models in predicting foregoing preventive dental care, applying SMOTE.

	Standard threshold[a]					Group-specific threshold[b]			
	AUROC (95% CI)	Sensitivity	Specificity	Precision[c]	F1[d]	Sensitivity	Specificity	Precision[c]	F1[d]
**Extreme Gradient Boosting**									
Asian	0.73 (0.69-0.78)	0.66	0.66	0.80	0.72	0.62	0.70	0.80	0.70
Black	0.74 (0.71-0.77)	0.91	0.36	0.83	0.87	0.79	0.57	0.86	0.82
Hispanic	0.76 (0.73-0.78)	0.89	0.43	0.85	0.87	0.80	0.59	0.87	0.83
White	0.84 (0.83-0.85)	0.78	0.78	0.81	0.80	0.75	0.80	0.82	0.78
Other[e]	0.73 (0.67-0.79)	0.81	0.51	0.81	0.81	0.69	0.70	0.86	0.77
18-64	0.80 (0.79-0.81)	0.84	0.63	0.82	0.83	0.72	0.75	0.85	0.78
≥65	0.88 (0.87-0.90)	0.84	0.81	0.84	0.84	0.80	0.83	0.85	0.83
High income	0.82 (0.81-0.83)	0.78	0.74	0.79	0.79	0.73	0.78	0.81	0.77
Low income (<200%	0.77 (0.75-0.79)	0.92	0.43	0.86	0.89	0.77	0.65	0.90	0.83
**Light Gradient Boosting**									
Asian	0.75 (0.70-0.79)	0.70	0.68	0.81	0.75	0.62	0.74	0.83	0.71
Black	0.74 (0.71-0.77)	0.91	0.38	0.83	0.87	0.77	0.58	0.86	0.81
Hispanic	0.77 (0.74-0.79)	0.89	0.47	0.85	0.87	0.73	0.70	0.89	0.80
White	0.84 (0.83-0.85)	0.78	0.77	0.80	0.79	0.75	0.80	0.81	0.78
Other[e]	0.70 (0.63-0.77)	0.83	0.44	0.80	0.82	0.79	0.53	0.82	0.80
18-64	0.80 (0.79-0.81)	0.84	0.63	0.82	0.83	0.72	0.75	0.85	0.78
≥65	0.88 (0.87-0.90)	0.84	0.81	0.84	0.84	0.80	0.83	0.85	0.83
High income	0.82 (0.81-0.83)	0.78	0.74	0.79	0.79	0.73	0.78	0.81	0.77
Low income	0.77 (0.75-0.79)	0.92	0.43	0.86	0.89	0.83	0.57	0.88	0.85
**Random Forest**									
Asian	0.77 (0.73-0.81)	0.74	0.71	0.84	0.79	0.37	0.92	0.90	0.53
Black	0.76 (0.74-0.89)	0.91	0.37	0.83	0.87	0.44	0.87	0.92	0.60
Hispanic	0.79 (0.76-0.81)	0.88	0.49	0.86	0.87	0.39	0.92	0.94	0.55
White	0.84 (0.83-0.85)	0.78	0.78	0.81	0.79	0.71	0.84	0.83	0.76
Other[e]	0.75 (0.69-0.81)	0.89	0.49	0.82	0.86	0.52	0.80	0.87	0.65
18-64	0.82 (0.81-0.83)	0.82	0.67	0.83	0.83	0.59	0.85	0.88	0.71
≥65	0.89 (0.87-0.90)	0.85	0.80	0.83	0.84	0.78	0.85	0.86	0.82
High income	0.83 (0.82-0.84)	0.80	0.78	0.75	0.79	0.70	0.81	0.83	0.76
Low income	0.80 (0.78-0.82)	0.91	0.46	0.87	0.89	0.44	0.89	0.91	0.60
**Catboost**									
Asian	0.72 (0.67-0.76)	0.71	0.65	0.80	0.75	0.49	0.83	0.85	0.62
Black	0.72 (0.68-0.75)	0.88	0.41	0.83	0.86	0.69	0.63	0.86	0.77
Hispanic	0.75 (0.72-0.77)	0.87	0.46	0.85	0.86	0.67	0.70	0.89	0.76
White	0.84 (0.82-0.85)	0.79	0.76	0.79	0.79	0.75	0.79	0.80	0.78
Other[e]	0.68 (0.62-0.75)	0.81	0.47	0.80	0.80	0.81	0.71	0.56	0.76
18-64	0.81 (0.79-0.82)	0.83	0.64	0.82	0.83	0.70	0.78	0.86	0.77
≥65	0.87 (0.86-0.89)	0.84	0.79	0.83	0.83	0.80	0.83	0.85	0.82
High income	0.82 (0.81-0.83)	0.78	0.73	0.79	0.78	0.73	0.78	0.80	0.77
Low income	0.77 (0.75-0.79)	0.90	0.48	0.87	0.88	0.69	0.72	0.90	0.78

[a]Standard threshold: 0.5. [b]Group-specific threshold was defined according to the prevalence of foregoing preventive dental care for each subgroup in the training dataset. For the full sample: 0.66; White: 0.56; Black: 0.78; Hispanic: 0.79; Asian: 0.62; Other or multiple races: 0.72; 18-64: 0.68; 65+: 0.60; high income: 0.58; and low income: 0.80. [c]Precision: Positive predictive value. [d]F1 score: harmonic mean of sensitivity and precision. [e]"Other" includes groups that were too small to be evaluated individually, such as American Indians and Alaska Natives, or those reporting multiple racial groups.

© 2023 Schuch HS et al. *JAMA Network Open.*
