# Supplementary material for: Fairness of Machine Learning Algorithms for Predicting Foregone Preventive Dental Care for Adults
Source: JAMA Netw Open. 2023 Nov 3;6(11):e2341625. doi: 10.1001/jamanetworkopen.2023.41625 (PMC10625037; doi:10.1001/jamanetworkopen.2023.41625)
Supplement: Supplement 2. — Data Sharing Statement [file jamanetwopen-e2341625-s002.pdf]

Data Sharing Statement

Schuch. Fairness of Machine Learning Algorithms for Predicting Foregone Preventive Dental Care for Adults. *JAMA Netw Open*. Published November 06, 2023. doi:10.1001/jamanetworkopen.2023.41625

Data
Data available: Yes
Data types: Deidentified participant data
How to access data: Deidentified data, data dictionary and codes will be publicly available on GitHub and/or Harvard Dataverse with the manuscript publication.
When available: With publication

Supporting Documents
Document types: Statistical/analytic code
How to access documents: We used deidentified publicly available data from the MEPS survey. Our supplemental material includes the data derivations detail and variable coding (etable 7). We provided a Data Sharing Statement in the revised version of the manuscript
When available: With publication

Additional Information
Who can access the data: Publicly available.
Types of analyses: Any purpose.
Mechanisms of data availability: Publicly available on MEPS website
